# Reliability of skeletal muscle ultrasound in critically ill trauma patients

**DOI:** 10.5935/0103-507X.20190072

**Published:** 2019

**Authors:** Luciana Vieira, Lara Patrícia Bastos Rocha, Sunita Mathur, Larissa Santana, Priscilla Flávia de Melo, Vinicius Zacarias Maldaner da Silva, João Luiz Quaglioti Durigan, Gerson Cipriano Jr.

**Affiliations:** 1 Programa de Pós-Graduação em Ciências e Tecnologias em Saúde, Universidade de Brasília - Brasília (DF), Brasil.; 2 Departamento de Fisioterapia, Hospital de Base do Distrito Federal - Brasília (DF), Brasil.; 3 Unidade de Terapia Intensiva, Hospital de Base do Distrito Federal - Brasília (DF), Brasil.; 4 Departamento de Fisioterapia, Universidade de Toronto - Toronto, Ontario, Canadá.; 5 Programa de Pós-Graduação em Ciências da Reabilitação, Departamento de Fisioterapia, Universidade de Brasília - Brasília (DF), Brasil.

**Keywords:** Trauma/diagnostic imaging, uadriceps muscle/diagnostic imaging, Emergency department, Muscular atrophy/diagnostic imaging, Ultrasonography, Diagnostic techniques and procedures

## Abstract

**Objective:**

To evaluate the safety and feasibility of the ultrasound assessment of quadriceps in the emergency setting. To assess the intra- and interrater reliability for the acquisition and analysis of ultrasound images of muscle thickness and echogenicity in critically ill trauma patients between health professionals with different levels of expertise.

**Methods:**

Diagnostic accuracy study. Two examiners (expert and novice) acquired ultrasound images from ten patients; an experienced, blinded analyst quantified the images. In a separate group of ten patients, two analysts (expert and novice) quantified quadriceps muscle thickness and echogenicity (square or trace method) from images acquired by one examiner.

**Results:**

Excellent reliability was found for image acquisition and analysis (intraclass correlation coefficients > 0.987; p < 0.001). The standard error of the measurement values ranged from 0.01 - 0.06cm for muscle thickness and from 0.75 - 2.04 arbitrary units for muscle echogenicity. The coefficients of variation were < 6% for thickness and echogenicity. The echogenicity values were higher when using the square technique than when using the tracing technique (p = 0.003).

**Conclusion:**

Ultrasound is safe, feasible, and reliable for muscle assessment in critically ill trauma patients, regardless of the assessor's level of expertise.

## INTRODUCTION

Major trauma is the leading cause of death and disability in young adults with no previous medical history,^(1)^ with a mortality rate of 30 to 70%, and the recovery of survivors is marked by persistent neurological sequelae, impaired functional status, and reduced quality of life.^(2)^ Additionally, changes in skeletal muscle thickness and echogenicity occur early and rapidly in critically ill patients^(3)^ and are associated with decreased muscle strength and functional outcomes.^(4)^ Currently, the demand for intensive care beds far exceeds their availability in many countries, and the shortage of beds in the intensive care unit (ICU) is an increasingly common phenomenon worldwide.^(5,6)^ Many polytrauma patients start critical illness support, such as invasive mechanical ventilation, at the Emergency Department (ED) and stay there for many hours, sometimes many days,^(7,8)^ highlighting the relevance of starting muscle assessment as early as ED admission.^(9)^

The evaluation of skeletal muscle atrophy typically requires high-cost, sophisticated imaging techniques such as dual-energy X-ray absorptiometry (D-XA),^(10)^ computed tomography, magnetic resonance imaging, or invasive methods such as muscle biopsy. These are challenging to conduct in trauma patients and are not routinely available in the emergency or critical care setting.^(11)^ Additionally, the diagnosis of muscle weakness, which is traditionally performed using volitional strength testing (e.g., Medical Research Council sum score),^(12)^ is delayed in mechanically ventilated patients due to sedation and inability to follow commands.^(13)^ Ultrasound is a noninvasive method for evaluating skeletal muscle thickness and echogenicity.^(14,15)^ Sonographic measurement of peripheral muscle thickness has been validated in healthy subjects^(16,17)^ Ultrasound provides a clinical utility for assessing the trajectory of change in skeletal muscle structure during critical illness.^(18,19)^ In a recent study, muscle echogenicity was associated with decreased muscle strength and functional capability at awakening in critically ill patients,^(4)^ suggesting that echogenicity could be used as a prognostic marker while the patient is not able to perform volitional tests.

Adequate reliability, a psychometric property reflecting the degree to which a measurement is consistent and free from error, is critical to any measurement and is a prerequisite for using measurements to make proper decisions regarding patients. ^(20)^ The reliability of skeletal muscle ultrasound in critically ill patients is not fully established. ^(21)^ Prior studies that evaluated the reliability of quantitative sonographic assessment of muscle thickness^(22,23)^ and echogenicity^(14,23)^ have been conducted only in the ICU, a more stable environment than the ED. Additionally, these studies focused only on image analysis; however, the acquisition of the images needs to be investigated since, in clinical practice, it is not always possible for the same assessor to acquire all the images.

Thus, this study was designed to (i) evaluate the safety and feasibility of ultrasound assessment of quadriceps at the ED and (ii) assess the intra- and interrater reliability of the acquisition and analysis of ultrasound images of muscle thickness and echogenicity in critically ill trauma patients among health professionals with different levels of expertise. Preliminary findings of this study were previously reported in the form of an abstract.^(24)^

## METHODS

This diagnostic accuracy study was conducted in a Level-I Trauma Center of a public hospital in Brasilia, Brazil, after obtaining institutional ethical committee approval (CAAE 19036013.8.0000.5553) and following the Helsinki Declaration of 1975. Written consent was obtained from the closest responsible family member since all patients were intubated and sedated at the time of enrollment. Ultrasound images were acquired in the ED within the first 24 hours of hospital admission after medical stabilization of the patient. Subjects were included between April and June 2014. The images were deidentified and analyzed between August and October 2015.

The sample size was determined according to the recommendations of Walter et al.,^(25)^ which recommend its analysis utilizing the intraclass correlation coefficients (ICCs). Considering the minimally acceptable reliability coefficient (ICC = 0.70), the expected reliability (ICC = 0.93, based on a similar study),^(23)^ a type I and type II error rates of (α = 0.05 and β = 0.20), to perform a three repetitions task, the recommend number was 9. We recruited 10 patients per group to compensate for potential attrition and unusable data.

Subjects were considered for inclusion in the study if they were admitted to the ED with major trauma, were more than 18 years of age, and were mechanically ventilated. Exclusion criteria were pregnancy, previous stroke, known a pre-existent neuromuscular disease, lower limb amputation, fracture or skin lesion that prevented ultrasound evaluation, or clinical suspicion of brain death.

The sample consisted of two groups of patients. Group 1 (n = 10) was used to test the reliability of image acquisition by two different examiners: one expert, a board-certified radiologist; and one novice, a health professional with no prior ultrasound imaging experience. A single, blinded analyst analyzed the images. Group 2 (n = 10) was used to test the reliability of image analysis. One examiner acquired all images, which were later quantified by two analysts with different levels of experience: one expert, with experience in image analysis; and one novice, with no previous experience.

The safety of the ultrasound evaluation was examined by recording the variability in cardiovascular and respiratory bedside parameters. Patients were continuously monitored throughout the assessment. A clinician observing the protocol recorded adverse events and timed the assessment. The following adverse events were recorded: changes of over 20% of the resting cardiovascular or respiratory parameters during positioning of the patient for assessment; accidental dislodgement or removal of drains, tubes, catheters, or vesical probes; self-extubation, accidental extubation or tracheostomy tube removal; and falling off the bed.

For muscle ultrasound acquisition, an experienced sonographer conducted a 20-minute training session using the same instructions for both examiners, as described in a previous study.^(26)^ An initial technical explanation about the protocol and a supervised performance in three patients was performed before commencing the evaluation of study patients. Acquisition settings (frequency, depth, and gain) were explained during the training session and were kept constant between examiners. For image analysis, the two assessors were instructed about basic settings of the software, region-of-interest placement, and identification of the anatomical structures (superficial fat, fascia, muscle, and bone).

Ultrasound images were acquired with a SonoSite M-Turbo^®^ portable ultrasound device (Sonosite, Inc., Bothell, WA, USA) equipped with a 2-dimensional, high-frequency linear array transducer (L38xi, bandwidth: 10 - 5MHz, maximal scan depth: 9cm). All images were acquired with a standardized protocol for transducer placement, anatomic landmarks, and patient position, based on previous studies.^(23,27)^ Subjects were assessed in the supine position with their knee in passive extension and neutral rotation.

A water-soluble transmission gel was applied to the ultrasound probe to allow acoustic contact without depressing the dermal surface. The probe was oriented transverse to the muscle length and perpendicular to the long axis of the thigh (transverse or axial plane) on its anterior surface, 50% of the distance from the anterior superior iliac spine to the superior patellar border. The transverse orientation of the probe was chosen because one previous study suggested that the cross-sectional sonographic view might be more sensitive to changes in muscle echogenicity in critically ill patients than the longitudinal view.^(27)^

All images were acquired at the same time of day and were obtained independently by each examiner to ensure blinding. The order of acquisition by the expert or novice was randomized for each patient. Three images per subject were obtained from the dominant thigh of each subject using a depth of 5.9cm. The images were saved and transferred onto a computer in a JPEG format for further analysis.

**Figure 1:**
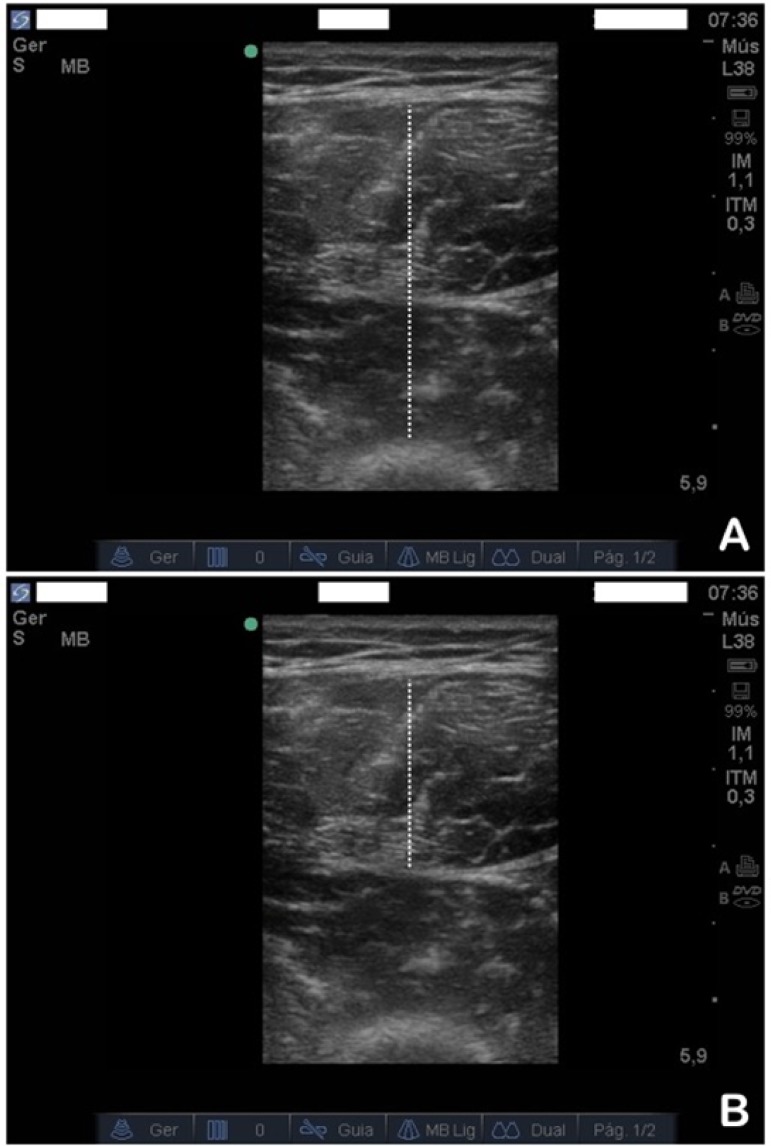
Example of image processing to measure muscle thickness. The dotted line represents total quadriceps thickness on panel (A) and *rectus femoris* thickness on panel (B).

ImageJ^®^ (NIH, Bethesda, MD, USA), an open-access software package, was used for image analysis. Three images per subject were quantified, and the average was used as a final value. Muscle size was analyzed using 2 measurements: (i) total quadriceps thickness (*rectus femoris* and *vastus intermedius* complex),^(27)^ defined as the maximum distance between the subcutaneous tissue and the bone surface of the femur, and (ii) *rectus femoris* thickness,^(4)^ measured from the subcutaneous tissue to the deep muscle aponeurosis (Figure 1). All measures of muscle thickness were expressed in centimeters (cm).

**Figure 2:**
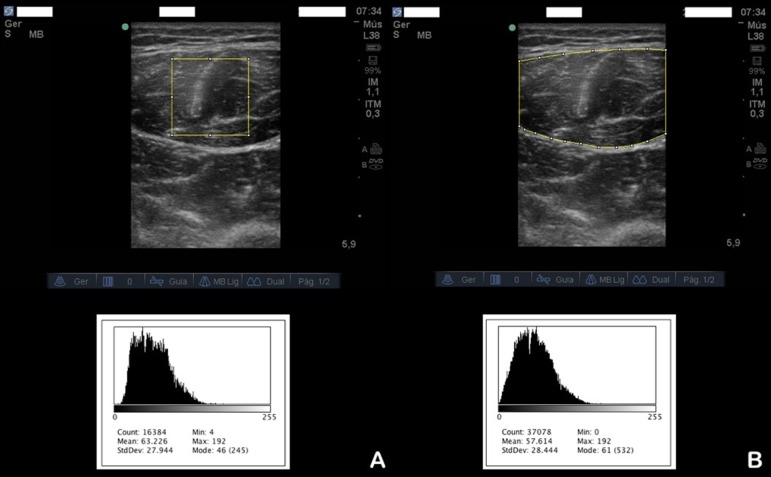
Example of image processing to measure muscle echogenicity. The selected area represents the region of interest, using (A) the square method or (B) the trace method; the grayscale is defined by the histogram below each image.

Muscle echogenicity was assessed by *rectus femoris* echogenicity (Figure 2) using a computer-assisted quantitative grayscale analysis. Two methods were used for outlining the region of interest: the trace method,^(28)^ in which the assessor highlighted all visible muscle area, avoiding the bone and surrounding fascia, for defining the region of interest, and the square method,^(27)^ in which a standard square area of 2 x 2cm was used to determine the region of interest (if the area to be analyzed was smaller than 2 x 2cm, the largest possible square within the anatomic boundaries of the *rectus femoris* muscle was examined). The mean echogenicity of the region of interest was calculated by using the histogram function of the software and expressed as a value between 0 (= black) and 255 (= white) in arbitrary units (AU).

### Statistical analysis

All statistical analyses were performed using Statistical Package for Social Science (SPSS) for Mac (version 21, IBM, Chicago, Illinois, USA). Data were tested for normality using the Shapiro-Wilk test (α = 0.05). Muscle thickness and echogenicity are expressed as the mean ± standard deviation (SD). Bland-Altman plots were also used to assess bias between sessions. Intra- and interrater reliability were determined by calculating the ICC with 95% confidence intervals using the average of the measurements on a scale of 0 to 1, where 1 represents perfect reliability, and zero represents an absence of association.^(29)^

The standard error of the measurement (SEM) was calculated to determine the typical error associated with the thickness or echogenicity measurement from ultrasound images captured between data collection sessions:

$$\mathrm{SEM}=\sqrt{\mathrm{SStotal}÷(n-1)}\times \sqrt{1-\mathrm{ICC}}$$

Where *SStotal* represents the overall variance in the model and *n* represents the total number of data sets.^(29)^

The mean coefficient of variation (CV) between examiners was also calculated. Differences between the two examiners and the two techniques were assessed by analysis of variance (ANOVA). A p-value of less than 0.05 indicated statistical significance.

## RESULTS

### Patient characteristics

Twenty mechanically ventilated trauma patients were consecutively recruited from the Trauma Unit in the ED (n=10 patients for image acquisition and n=10 for image analysis). Table 1 shows the baseline characteristics of the subjects included.

**Table 1:** Demographic and clinical characteristics of the study sample

Characteristics	Acquisition	Analysis
Age (years)	35.4 ± 12.5	37.1 ± 13.4
Male sex	8 (80)	8 (80)
APACHE II score	15.8 ± 3.3	16.2 ± 2.7
BMI (kg/m^2^)	25.7 ± 8.3	24.9 ± 6.8
Injury severity score	38.5 ± 18.0	39.4 ± 15.3
Sepsis	7 (70)	6 (60)
Septic shock	3 (30)	4 (40)
Multiorgan failure	3 (30)	4 (40)

APACHE - Acute Physiologic and Chronic Health Evaluation; BMI - body mass index. p > 0.05 for all comparisons between groups. Results expressed as the mean ± standard deviation or n (%).

### Safety and feasibility

We observed minor oscillations in oxygen saturation, respiratory rate, and heart rate related to positioning patients during ultrasound evaluation, within established safety limits. No significant safety or adverse events related to catheters, accidental extubation, or falling from a bed occurred. Image acquisition took less than 10 minutes per patient.

### Reliability

The intrarater reliability was excellent for both muscle acquisition (ICCs > 0.952) and analysis (ICCs > 0.988), despite the level of expertise of the assessor (Table 2). Similarly, the interrater reliability between-assessors was also excellent for both muscle acquisition (ICC > 0.977) and analysis (ICC > 0.961), despite the level of expertise. The SEM values ranged from 0.01 to 0.06cm for muscle thickness and from 0.75 to 2.04 AU for muscle echogenicity.

**Table 2:** Intrarater reliability for three acquired or analyzed images of muscle thickness and echogenicity by assessor

Parameter	Expert	Novice
Acquisition (n = 10)		
Thickness - quadriceps (cm)	0.952 (0.821 - 0.988)	0.999 (0.998 - 1.000)
Thickness - *rectus femoris*(cm)	0.999 (0.997 - 1.000)	0.997 (0.988 - 0.999)
Echogenicity - square (AU)	1.000 (0.999 - 1.000)	0.996 (0.984 - 0.999)
Echogenicity - trace (AU)	0.999 (0.995 - 1.000)	0.987 (0.949 - 0.997)
Analysis (n = 10)		
Thickness - quadriceps (cm)	0.999 (0.996 - 1.000)	1.000 (0.999 - 1.000)
Thickness - *rectus femoris*(cm)	0.998 (0.994 - 1.000)	0.998 (0.992 - 1.000)
Echogenicity - square (AU)	0.999 (0.998 - 1.000)	0.997 (0.989 - 0.999)
Echogenicity - trace (AU)	0.999 (0.997 - 1.000)	0.988 (0.954 -0.997)

AU - arbitrary units. Results expressed as the intraclass correlation coefficients (95% confidence interval).

**Figure 3:**
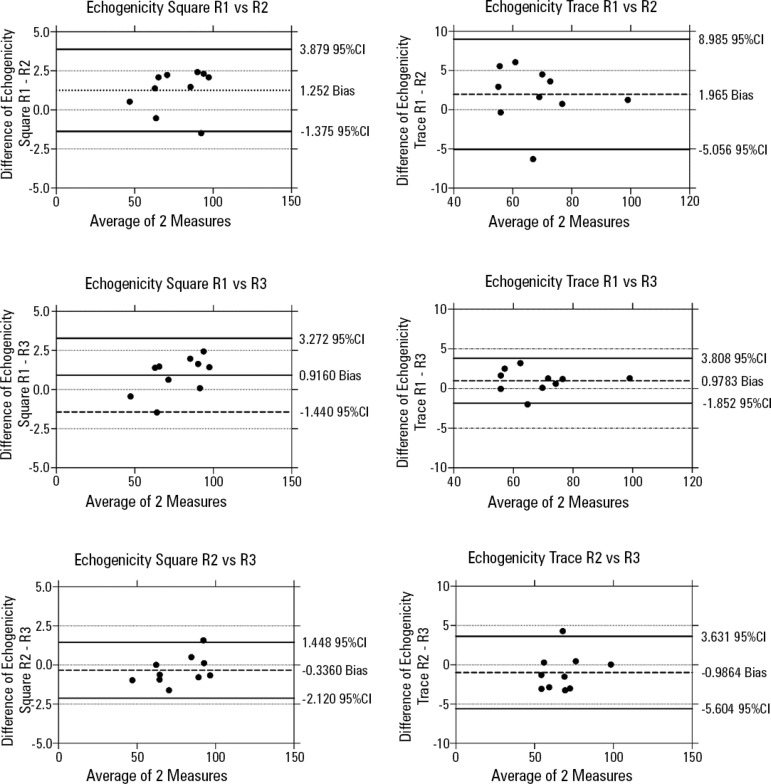
Bland-Altman plots using for concordance of the inter-rater evaluations between the evaluation of the echo square and echo traced. 95%CI - 95% confidence interval.

**Figure 4:**
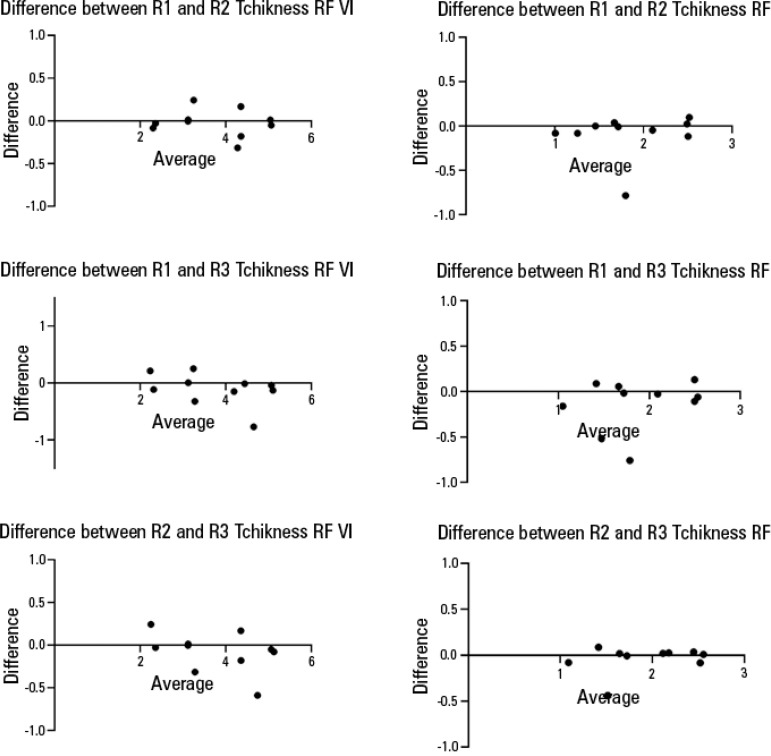
Bland-Altman plots for the concordance of the interrater evaluations of the thickness *rectus femoris vastus intermedius* and thickness *rectus femoris*. RF - *rectus femoris*. VI - *vastus intermedius*.

For both the image acquisition and analysis by different assessors, the CV for muscle thickness was lower for the total quadriceps thickness than for the *rectus femoris* thickness. For echogenicity, the CV was lower for the square technique than for the tracing technique (Table 3). There were no significant differences in muscle thickness or echogenicity between assessors with different levels of expertise (p > 0.05), as presented in table 4. Bland-Altman plots for each condition showed no bias in either measurement (echo intensity or thickness) among three assessors (Figures 3 and 4). However, echogenicity values were significantly higher when quantified by the square technique compared to the tracing technique (p < 0.001) for both image acquisition and analysis.

**Table 3:** Interrater reliability for image acquisition and analysis of muscle thickness and echogenicity

Parameter	ICC	SEM	CV
Acquisition (n = 10)			
Thickness - quadriceps (cm)	0.990 (0.970 - 0.997)	0.06	2.55
Thickness - *rectus femoris*(cm)	0.990 (0.964 - 0.997)	0.04	5.69
Echogenicity - square (AU)	0.996 (0.986 - 0.999)	1.73	1.29
Echogenicity - trace (AU)	0.977 (0.934 - 0.994)	0.75	2.31
Analysis (n = 10)			
Thickness - quadriceps (cm)	0.999 (0.997 - 1.000)	0.02	1.55
Thickness - *rectus femoris*(cm)	0.999 (0.997 - 1.000)	0.01	4.57
Echogenicity - square (AU)	0.994 (0.968 - 0.999)	2.04	2.54
Echogenicity - trace (AU)	0.961 (0.894 - 0.989)	1.02	3.99

ICC - intraclass correlation coefficient; SEM - standard error of the measurement; CV - coefficient variation; AU - arbitrary units. Data are presented as intraclass correlation coefficients (95% confidence intervals) or %.

**Table 4:** Absolute values of muscle thickness and echogenicity for acquisition and analysis by two assessors with different levels of expertise.

Parameter	Expert	Novice	p value
Acquisition (n = 10)			
Thickness - quadriceps (cm)	3.43 ± 0.87	3.47 ± 0.84	0.97
Thickness - *rectus femoris*(cm)	1.86 ± 0.55	1.82 ± 0.52	0.96
Echogenicity - square (AU)	77.4 ± 16.8	76.4 ± 16.7	0.98
Echogenicity - trace (AU)	69.1 ± 12.7	67.5 ± 16.1	0.94
Analysis (n = 10)			
Thickness - quadriceps (cm)	3.18 ± 0.85	3.16 ± 0.88	0.99
Thickness - *rectus femoris*(cm)	1.67 ± 0.48	1.72 ± 0.50	0.98
Echogenicity - square (AU)	83.9 ± 18.6	81.8 ± 17.6	0.96
Echogenicity - trace (AU)	67.7 ± 14.6	65.1 ± 14.4	0.94

AU - arbitrary units. A between-measures analysis of variance was used to test for significant differences between assessors and techniques; no significant differences were found for muscle thickness or echogenicity between assessors with different levels of expertise or between techniques for muscle thickness; however, echogenicity was significantly higher when quantified by the square technique compared to the tracing technique, for both acquisition and analysis (p < 0.001). Results expressed as the mean ± standard deviation.

## DISCUSSION

Based on our findings, the assessment of muscle thickness and echogenicity by ultrasound in the first 24 hours after hospital admission to the ED is safe and feasible in critically ill trauma patients, with excellent reproducibility for both image acquisition and analysis. Ultrasound images were acquired in less than 10 minutes per patient, corroborating some previous studies in a critical care setting.^(4,22)^ Our results confirm that it is possible to standardize these measurements between assessors with different levels of expertise with a brief 20-minute training session, as previously demonstrated in healthy subjects.^(26)^ We found that echogenicity was significantly higher when quantified by the square technique compared to the tracing technique. The use of the square technique to select the region of interest to quantify echogenicity resulted in a smaller CV than the use of the tracing technique, suggesting that the square technique should be chosen for echogenicity analysis.

The impact of critical illness on the musculoskeletal system has been reported since 1984.^(30)^ Muscle wasting is common and is associated with longstanding consequences that dramatically affect recovery;^(31)^ it occurs early and rapidly during the first week after ICU admission.^(3)^ Moreover, it may precede admission to the ICU, beginning earlier than previously demonstrated, right after hospital admission to the Emergency Room. Even in healthy young subjects, short periods of muscle disuse lead to substantial loss of skeletal muscle mass, accompanied by an early catabolic molecular signaling response.^(32)^ Changes in muscle echogenicity in subjects with acute conditions can occur early and might be detected easily,^(27)^ which increases the relevance of these measurements for critically ill trauma patients starting in the emergency setting.

A recent retrospective study^(33)^ demonstrated that low muscle mass at ICU admission was an independent predictor of mortality and was associated with increased disability and a higher frequency of discharge to a nursing home. In addition, it has been demonstrated that patients with the greatest amount of muscle on admission to the ICU lost significantly more muscle thickness than those with thinner muscles.^(34)^ However, it remains unclear whether the change in muscle size from baseline or the absolute muscle size on admission is the most important predictor of functional outcome and mortality.^(35)^ Additionally, it is essential to standardize operating protocols and to assure adequate reliability for acquisition and analysis of the images,^(36)^ this allows clinicians to make informed decisions about the care of patients and enables better understanding, comparison, and meta-analysis of data from different studies.^(20)^

The potential variability in image acquisition by different evaluators has been suggested as a significant factor impeding the widespread use of ultrasound in clinical and research settings. Our study utilized two health professionals, an expert, and a novice, with different levels of expertise in ultrasound utilization, after a 20-minute training session. Even with minimal training, our results showed excellent values for the ICC and CV, demonstrating the potential for ultrasound to be incorporated into routine clinical care.

Furthermore, the images were all obtained in less than ten minutes per patient. Corroborating our findings, a recent study^(23)^ in critically ill patients observed excellent interrater reliability within novices and experienced examiners. Zaidman et al.^(26)^ also reported that minimal training was required to reliably perform muscle ultrasound in healthy boys and boys with Duchenne muscular dystrophy.

There is a growing interest in the measurement of changes in muscle echogenicity, particularly in critically ill patients who experience rapid disuse atrophy as well as muscle edema. A strong correlation between echogenicity and measurements of fibrosis and intramuscular fat from muscle biopsies has also been demonstrated in critically ill patients.^(37)^ Subjective evaluation of muscle ultrasound using a visual qualitative scale showed relatively low interrater agreement, which further deteriorated when an inexperienced observer interpreted the images. For this reason, computer-aided quantitative techniques have been introduced to improve objectivity in image interpretation.^(38)^ Quantification of muscle echo intensity can be achieved with grayscale analysis.^(18)^

Differences in system settings, such as increased gain, can give muscles a whiter appearance that can be mistaken for pathologically increased echo intensity;^(39)^ this highlights the importance of standardized protocols. Additionally, different techniques for delineating the region of interest may lead to different values of echogenicity, as demonstrated in our study. For Sarwal et al.,^(23)^ part of the discrepancy between the square and tracing techniques might be a result of the variability generated during the selection of the region of interest; with the tracing technique, the selected area might include the intermuscular connective tissue, fascia, and blood vessels. Restriction to a predefined square focused mostly on muscular bulk will have a higher consistency than other regions of interest. Due to the small CV, the selection of the region of interest by the square technique may be preferable in critically ill patients.

Our study has some limitations. The evaluation of muscle size and cross-sectional area of the *rectus femoris* has been described in critical care patients;^(27,39)^ however, the visualization of all edges of the muscle is not possible at the mid-thigh in some patients without an extended field of view imaging,^(28)^ which was not available with our system. We did not evaluate between-day agreement because rapid muscle wasting within 48 hours has already been demonstrated in critically ill patients^(4)^ and would interfere in the analysis.

## CONCLUSIONS

In conclusion, muscle ultrasound represents an attractive modality in different critical care settings, as it was safe and applicable in critically ill trauma patients in the Emergency Department. The ultrasound protocol presented excellent intra- and inter reliability for both image acquisition and analysis after a brief 20-minute training session, regardless of the assessor's level of expertise, and could be used in future studies evaluating longitudinal changes.
